# Disparities in the Epidemiology of Anal Cancer: A Cross-Sectional Time Series

**DOI:** 10.1089/heq.2020.0021

**Published:** 2020-09-16

**Authors:** Carlos R. Oliveira, Yu S. Niu, Hulda M. Einarsdottir, Linda M. Niccolai, Eugene D. Shapiro

**Affiliations:** ^1^ Department of Pediatrics, Yale School of Medicine, New Haven, Connecticut, USA.; ^2^ Department of Surgery, Yale School of Medicine, New Haven, Connecticut, USA.; ^3^ Department of Epidemiology of Microbial Diseases, Yale School of Public Health, New Haven, Connecticut, USA.

**Keywords:** anal cancer, disparities, HPV vaccine, trends

## Abstract

**Purpose:** To assess the trends and sociodemographic disparities of anal cancer.

**Methods:** For this time series, billing claims were reviewed for all encounters between 2007 and 2011 in the Yale New Haven Health System.

**Results:** There were 80 new cases identified. Decreasing trends were seen in women and increasing trend in men (−30.1% and 27.3%). Diagnoses were more common in areas with the highest proportion of racial minorities (incidence rate ratio [IRR]=1.75; *p*≤0.01) and poverty (IRR=1.72; *p*=0.04).

**Conclusions:** Anal cancer continues to rise in men during the postvaccine era. Communities with the highest proportion of poverty and racial/ethnic minority groups bear the highest burden of disease.

## Introduction

The incidence of anal cancer has been steadily rising in both men and women in the United States.^1^ Studies in several states have shown incidences in recent years that ranged between 2 and 7 cases per 100,000 in individuals aged 35–65 years.^2^ The U.S. Centers for Disease Control and Prevention estimate that >8000 cases of anal cancer have been diagnosed every year since 2015, most of which (>65%) occurred in women.^3^ In 2006, human papillomavirus (HPV) vaccines were licensed in the United States for use among females aged 9 to 26 years, primarily for the prevention of HPV infections and of cervical cancer. More than 90% of anal cancers are attributable to one of the nine HPV types targeted by the currently available HPV vaccines, which suggests that anal cancer is largely vaccine preventable.^4^ In 2011, the United States became the first country to include males 9–21 years of age in their routine HPV vaccine immunization program.^5^ It is unknown whether this universal immunization strategy will reduce the rising epidemic of anal cancer in men as well as in women.

During the pre-HPV vaccine era, studies found that the rates of incidence and mortality of HPV-associated diseases disproportionally affected individuals with low incomes and racial/ethnic minority groups.^6,7^ Now that the HPV vaccines have been introduced, it is important to conduct surveillance of the incidence of anal cancer to ensure that they are achieving their fullest potential by reducing the incidence of anal cancer and its associated health disparities. The objectives of this study were to provide baseline estimates of the incidence of anal cancer diagnoses over time and to assess for differences in the incidence of disease by zip-code-based sociodemographic characteristics.

## Methods

For this cross-sectional time series, we reviewed clinical encounter claims from the Yale-New Haven Health System (YNHHS) to identify incident cases of anal cancer among residents of New Haven County, a racially and economically diverse metropolitan area. Encounter-specific discharge diagnosis codes were electronically extracted from both the inpatient and the outpatient records of residents of New Haven County who received care between January 1, 2007 and December 31, 2011 at a Yale-affiliated facility. Cases of anal cancer were identified using the ninth revision of the International Classification of Disease (ICD-9) system code for a malignant neoplasm of the anus or anal canal (codes: 154.2–154.8). To limit the analysis to incident cases, only the first mention of anal cancer in patients with multiple diagnostic codes during the study period was included.

For the primary analysis, we estimated the annual incidence rate and the mean percentage change in the incidence of anal cancer using Poisson regression. In these models, the number of cases of anal cancer within each zip-code was the dependent variable, and census-derived population estimates were used as offsets (on a log scale) to account for the sociodemographic variability within each zip-code. The trends of diagnosis were estimated by fitting the incidence of anal cancer to Poisson regression using the calendar year as a regressor. *p*-Values assessed whether the slope of the fitted line was significantly different than 0. *p*-Values <0.05 were considered statistically significant for all comparisons. For analyses of health disparities, zip-code-based sociodemographic characteristics (e.g., the proportion of residents who are living below federal poverty level, racial/ethnic minorities, college educated, or unemployed) were extracted for each zip-code in New Haven County from the American Community Survey data set.^8^ Analyses were conducted between August 2018 and January 2020 using Stata statistical software 15.0 (StataCorp, College Station, TX). This protocol was approved by the institutional review board of Yale University.

## Results

**FIG. 1.**
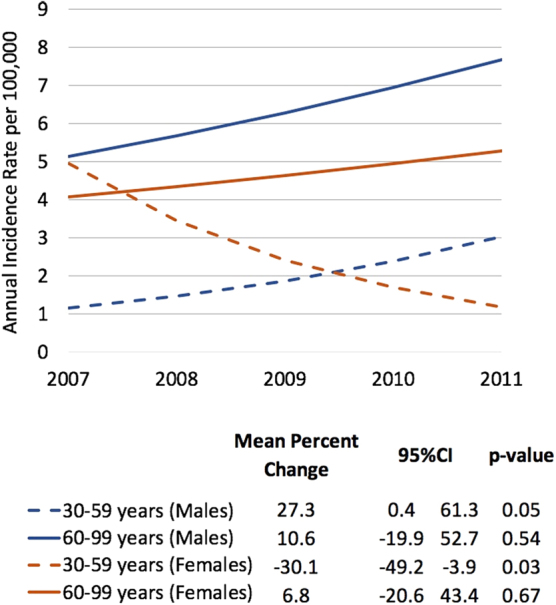
Annual incidence of anal cancer by sex and age. Incidence rate of anal cancer per 100,000 population between 2007 and 2011 in New Haven County, stratified by sex (male and female) and age group (30–59 and 60–99 years).

A total of 120 new cases of anal cancer were identified in the YNHHS clinical encounter claims data, of whom 80 were residents of New Haven County and were included in this analysis (median age=58 years, interquartile range=51–67 years). Most cases were women (48 patients [60.0%]) and were either privately or publicly insured (23 and 39 patients [35.4 and 60.0%], respectively). Over the 5-year period, the overall incidence was higher in women than in men, although this difference was not statistically significant (incidence rate ratio [IRR]=1.39, *p*=0.49). The mean age and sex-adjusted incidence rate were 4.47 cases per 100,000 population (95% confidence interval [CI]: 3.24 to 6.16). The mean percentage change for men (19.0; [95% CI: −6.9 to 52.1]) was substantially higher than that for women (−13.7 [95% CI: −36.9 to 18.1]). Stratifying by both age group and sex, we identified several distinct trends (Fig. 1). Statistically significant decreases were seen in the rates of anal cancer in younger women (30–59 years: mean percentage change=−30.1); however, increases were noted in the rates in younger men (30–59 years: mean percentage change=27.3). The rates of anal cancer varied by zip-code-based sociodemographic characteristics and are given in Table 1. Cases of anal cancer were more likely to occur in zip-codes with the highest proportion of racial minority groups (≥50%; IRR=1.75; *p*≤0.01), and the highest proportion of individuals with incomes below the poverty threshold (≥25%; IRR=1.72; *p*=0.04). No association was seen between the rates of anal cancer and the other measured zip-code-based sociodemographic variables (i.e., proportions that were unemployed or college educated).

**Table 1.** Incidence of Anal Cancer per 100,000 Population Ages 30 to 99 Years by Socioeconomic Characteristics: New Haven County, 2007 to 2011

	Cases	Population^a^	IR	IRR	p
Individual-level characteristics					
Total	80	1,789,655	4.47	—	—
Age, years					
30–59	42	1,314,155	3.20	Reference	
60–99	38	475,500	7.99	2.50	<0.01^b^
Sex					
Male	32	860,515	3.72	Reference	
Female	48	929,140	5.17	1.39	0.49
Area-level characteristics					
Proportion in poverty^c^					
<10%	44	1,113,135	3.95	Reference	
10–24%	27	544,315	4.96	1.25	0.45
≥25%	9	132,205	6.81	1.72	0.04^b^
Proportion of minorities^d^					
<25%	40	1,036,470	3.86	Reference	
25–49%	22	487,235	4.52	1.17	0.68
≥50%	18	265,950	6.77	1.75	<0.01^b^
Proportion with some college					
<50%	59	1,367,710	4.31	Reference	
≥50%	21	421,945	4.98	1.15	0.62
Proportion unemployed					
<5%	30	777,400	3.86	Reference	
≥5%	50	1,012,255	4.94	1.27	0.33

^a^ Population counts for 21 zip-codes within New Haven County from the ACS Demographic and Housing Estimates.

^b^ *p*-Value <0.05 was considered statistically significant.

^c^ Based on the United States federal poverty line.

^d^ Black or Hispanic.

IR per 100,000 population (5-year average from 2007 to 2011).

ACS, American Community Survey; IR, incidence rate; IRR, incidence rate ratio.

## Discussion

In this analysis, we report estimates of the incidence of anal cancer diagnoses over time in a sociodemographically diverse population and add to the evidence of cancer-related disparities. Although, on average, a higher proportion of the new cases of anal cancer occurred in women, we found the incidence of anal cancer to be rising most rapidly in younger men (30–59 years). Highly efficacious HPV vaccines have been available in the United States for females since 2006 and for males since 2011.^5^ However, the impact of HPV vaccines on rates of anal cancer is not likely observable during this early study period, given the prolonged lag time between HPV infection and development of anal cancer. These data, however, provide a baseline for future studies that assess the vaccine's impact on the incidence of anal cancer.

Very little is known about what is driving the sex-related differences in the trends of anal cancer. Receptive anal intercourse and infection with human immunodeficiency virus (HIV) are by far the most important risk factors for developing anal cancer.^9,10^ Nelson et al.,^1^ and others,^11^ have speculated that changes in the patterns of sexual behavior among men who have sex with men, a growing subgroup of the population in the United States, could be an important driver of the rising rates of anal cancer among men. Several previous studies have also shown a strong correlation between both cervical dysplasia and anogenital warts and the subsequent development of anal cancer.^12,13^ It is possible that the reduction in cervical precancers and anogenital warts that have been reported for the past 20 years could be contributing to the observed decreasing trends of anal cancer in women.^14–16^

After analyzing the zip-code-specific patterns of disease, we also found significant sociodemographic differences in the incidence of anal cancer. The neighborhoods with the highest proportion of racial minority groups, and the highest number of individuals living in poverty, were disproportionately affected by anal cancer. The reason for this difference in incidence is not well understood. Various population-level factors (e.g., the prevalence of high-risk HPV types, and sexual mixing patterns in the community) and individual-level factors (e.g., sexual behaviors, access to screening, and treatment) could be directly influencing the individual's risk of developing disease.^11,17^ Future study is needed to better understand and mitigate the drivers of this spatiotemporal heterogeneity.

Our study has potential limitations. First, we used existing clinical encounter claims data, which could have led to misclassification of cases. However, a recent study estimated that compared with chart reviews, ICD-9 discharge diagnosis codes were relatively accurate (92% sensitive and 96% specific) at detecting incident cases of cancer.^18^ Second, we did not have access to data on race or ethnicity at the individual level. Thus, we used zip-code-based sociodemographic characteristics as a composite indicator of individual-level socioeconomic position. More research is needed to validate the inferences being made using these aggregate measures. Third, these data could be underestimating the true incidence of anal cancer, as some individuals may have opted to receive care at a site not affiliated with the YNHHS. However, to identify patients for our study, we used data from YNHHS, the largest health care provider in the state, with >2 million outpatient encounters every year. Furthermore, we included data from the sole comprehensive cancer center of the state, which is centrally located within our study population. Thus, the number of cases within our well-defined study population who were not captured in these analyses is likely to have been small. Last, it should also be noted that our sample was relatively small, and some of our estimates had wide CIs. However, our point estimates are consistent with those reported in several other studies.^1,2,11,17^

## Conclusions

These data show that although both men and women are developing anal cancer, the incidence in young adult males is climbing. Our data also suggest there are significant sociodemographic differences in anal cancer, wherein communities with a higher proportion of individuals living in poverty and a higher proportion of racial/ethnic minority groups bear the highest incidence of disease. Continued surveillance is needed to determine whether these trends will reverse as the uptake of the vaccines increases in both sexes.

## Abbreviations Used

ACS: American Community Survey
CI: confidence interval
HPV: human papillomavirus
ICD-9: ninth revision of the International Classification of Disease
IR: incidence rate
IRR: incidence rate ratio
YNHHS: Yale-New Haven Health System
